# Development and validation of a climate-based ensemble prediction model for West Nile Virus infection rates in *Culex* mosquitoes, Suffolk County, New York

**DOI:** 10.1186/s13071-016-1720-1

**Published:** 2016-08-09

**Authors:** Eliza Little, Scott R. Campbell, Jeffrey Shaman

**Affiliations:** 1 Department of Environmental Health Sciences, Mailman School of Public Health, Columbia University, New York, NY USA; 2 Arthropod-Borne Disease Laboratory, Suffolk County Department of Health Services, Yaphank, NY USA

**Keywords:** West Nile Virus, *Culex* spp., Ensemble Prediction Model

## Abstract

**Background:**

West Nile Virus (WNV) is an endemic public health concern in the United States that produces periodic seasonal epidemics. Underlying these outbreaks is the enzootic cycle of WNV between mosquito vectors and bird hosts. Identifying the key environmental conditions that facilitate and accelerate this cycle can be used to inform effective vector control.

**Results:**

Here, we model and forecast WNV infection rates among mosquito vectors in Suffolk County, New York using readily available meteorological and hydrological conditions. We first validate a statistical model built with surveillance data between 2001 and 2009 (m09) and specify a set of new statistical models using surveillance data from 2001 to 2012 (m12). This ensemble of new models is then used to make predictions for 2013–2015, and multimodel inference is employed to provide a formal probabilistic interpretation across the disparate individual model predictions. The findings of the m09 and m12 models align; with the ensemble of m12 models indicating an association between warm, dry early spring (April) conditions and increased annual WNV infection rates in *Culex* mosquitoes.

**Conclusions:**

This study shows that real-time climate information can be used to predict WNV infection rates in *Culex* mosquitoes prior to its seasonal peak and before WNV spillover transmission risk to humans is greatest.

**Electronic supplementary material:**

The online version of this article (doi:10.1186/s13071-016-1720-1) contains supplementary material, which is available to authorized users.

## Background

West Nile Virus (WNV), first introduced in North America in New York during 1999, quickly spread across the United States. Each year, human WNV cases peak during mid to late summer. Most infections are asymptomatic (~80 %); however, some result in flu-like symptoms (~20 %) and in rare cases people suffer neuroinvasive disease (< 1 %). More troubling and less understood are the links between acute WNV and chronic morbidity [1]. In the United States (US) there have been 18,810 cases of neuroinvasive disease (1641 deaths) and 22,952 cases of non-neuroinvasive disease (124 deaths) reported since 1999 [2]. More recently, cases of WNV neuroinvasive disease spiked during 2012 to numbers not observed since 2003, suggesting that WNV outbreaks will continue to be a problem in the US [2, 3]. Presently, there is no vaccine for WNV, so reduction of human-vector contact through mosquito control and behavioral measures remains the main means of preventing WNV transmission.

WNV in the US is maintained by an enzootic cycle driven by virus transmission between avian reservoir hosts and bird-biting mosquito vectors. To date, 65 mosquito species have been found infected with WNV in the US [4]; however, only a few of these species are likely important in the transmission of WNV. In the northeastern US, *Culex pipiens* and *Culex restuans* are the suspected enzootic vectors while *Culex pipiens* and *Culex salinarius* are the main epidemic vectors [5, 6]; however, the critical vector(s) may change over a season and may not be fully enumerated [6].

WNV has also been detected in 326 bird species in the US; however, like the mosquito vectors, only a few species significantly influence transmission dynamics by amplifying the virus [7, 8]. Humans do not develop high enough viremia in response to WNV infection to infect mosquitoes and thus are not involved in the spread of WNV. Instead, enzootic transmission and amplification of WNV is supported by the co-occurrence of amplifying avian reservoir hosts, mosquito vectors, and virus prevalence in the mosquito vector populations. Furthermore, favorable environmental conditions can foment this co-occurrence and virus amplification, increase the numbers of infected mosquitoes, and increase transmission risk to humans [9].

A number of physical environmental conditions have been shown to affect WNV transmission dynamics. Temperature influences the rate of vector development, vector biting behavior, viral replication in vectors, virus transmission efficiency to avian hosts, and the seasonal phenology of avian hosts [10, 11]. Overall, increased temperatures accelerate virus amplification and transmission. Standing water provides breeding sites for mosquitoes, but the influence of rainfall on vector population dynamics is not linear: while above average rainfall may lead to higher mosquito abundance, extreme rainfall events may reduce larval survival through flushing effects [12]. Below average rainfall, or drought, may facilitate the population growth of certain species due to reduced predation, and remnant wetlands in periods of drought may concentrate resources for both mosquito vectors and avian hosts facilitating WNV amplification within these populations. Consequently, local hydrological conditions can provide insight into water resource availability for both vector and host, and have been found predictive of WNV transmission dynamics [9, 13]. Humidity has also been positively correlated with the population dynamics of some vector species [10].

The extensive distribution of WNV is tied to its ability to persist in multiple mosquito vectors that in turn inhabit a wide variety of ecosystems. [14] The influence of climate on WNV transmission risk in the US varies by the geographic range of disease vectors [15–22]. Due to identified differences in WNV disease ecology, we focus on studies of WNV disease ecology in the northeastern US to inform our research in this area.

In the northeastern US most research either links climate and landscape variables to human WNV cases [15, 16, 18, 20, 23–29] or to vector abundance [19, 30–34] but there is limited research linking climate to WNV infected mosquitoes [33, 35–37]. The number of WNV infected *Culex* mosquitoes influences human transmission risk by increasing the frequency of contact between infected vectors and humans [5]. Therefore, WNV transmission to humans is directly related to the abundance of infectious mosquitoes [27, 38], and the advent of human cases arise from the underlying effects of climate and landscape on the WNV enzootic transmission cycle. In the northeastern US, above average temperature is linked to increased enzootic WNV transmission and risk of spillover to humans [19, 24, 28, 33, 35–37]. Studies have also linked vector abundance and WNV transmission with lower than average rainfall [15, 20, 21, 33, 36, 37]; however, the effects of rainfall over time has not been explored in depth.

Accordingly, this study aims to model and forecast WNV infection rates in mosquito vectors using readily available monthly mean meteorological and hydrological conditions between January and August to assess their temporal influence. Accurate model discrimination in space and time of areas at risk for mosquitoes with higher WNV infection rates can, in theory, be used to inform the allocation of limited resources for more effective vector control; however, such out-of-sample model prediction must be tested before being put into practice. Indeed, models built to explain vector-borne disease dynamics are not often validated with prospective data [39]. Therefore, a central aim of this study is the validation of forecasts generated in real time with prospective environmental data as it became available at monthly time steps.

To better understand the dynamics of WNV transmission, we here revisit a model describing the spatial-temporal distribution of positive *Culex* mosquito pools collected in Suffolk County, Long Island, New York. That statistical model (referred to as m09) used meteorological and hydrological conditions to simulate WNV infection in *Culex* mosquitoes during 2001–2009 [36]. Here, we use pooled *Culex* WNV infection data collected during 2010–2015 to validate m09 predictions. We then explore alternate models using a longer record (2001–2012) of mean meteorological and hydrological data to estimate annual *Culex* WNV infection data. We then use multimodel inference to identify dominant environmental predictors and develop a weighted ensemble prediction framework, which is used to make retrospective predictions for 2013 and 2015 as well as prospective, real-time predictions for 2014.

## Methods

### Study area

**Fig. 1:**
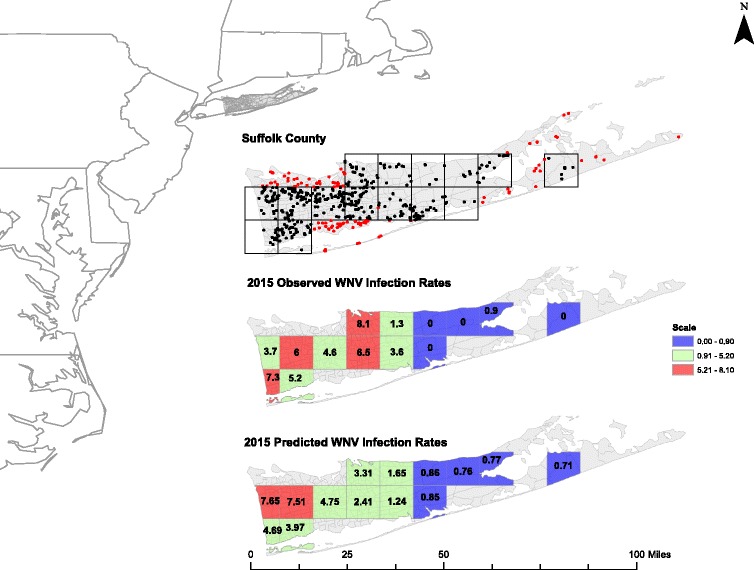
Map showing location of Suffolk County Long Island, trap locations within Suffolk County both those included and excluded for analysis, the scale of the NLDAS Grid Cells (13 km^2^), and both the observed and predicted 2015 WNV infection rates

Suffolk County occupies the eastern part of Long Island NY, roughly 15 miles east of New York City and covers an area of approximately 2370 mile^2^ (Fig. 1). The County is made up of densely populated residential and commercial properties in the west and is less populated with more agricultural and rural areas in the east.

### Mosquito pool data

This study uses WNV-assayed pools of *Culex* spp. mosquitoes. Mosquito collections were made throughout Suffolk County during 2001–2015 using both Centers for Disease Control and Prevention (CDC) gravid and light traps. Gravid traps were baited with rabbit-chow infusion and light traps with dry ice. Mosquito surveillance was conducted weekly from June to October, depending upon mosquito population levels and the presence of WNV in mosquitoes. Trap locations were guided by the historical presence of WNV at the beginning of the season and expanded based on the occurrence of WNV found in vectors and humans as the season progressed. Collected mosquitoes were anesthetized with dry ice and identified. *Culex pipiens* and *Culex restuans* have very similar morphology and, compounded by damage during the collection process, are often indistinguishable; consequently, these two species are grouped together for arboviral testing. *Culex salinarius* are separated whenever possible, but again, due to damage of identifying characteristics during collection are often included in *Culex pipiens/restuans* pools for testing. For arboviral analysis, pools were submitted to the New York State Department of Health (Arbovirus Laboratory, Wadsworth Center).

Table 1 presents a summary of the mosquito data including the number of trap locations, the underlying data for the derivation of vector abundance, percent of pools positive for WNV, and WNV infection rates. Vector abundance was calculated as the total number of all *Culex* mosquitoes captured divided by the total number of trap nights and provides a measure of the relative number of mosquitoes. High mosquito abundance may occur in the absence of infection and outbreaks may occur when abundance is low [14]. The percent of pools positive for WNV (PP) was calculated as the sum of positive pools divided by the sum of all pools and provides an estimate of the rate of WNV in mosquitoes tested. To measure infection rate, a maximum likelihood estimate (MLE) was calculated to estimate the prevalence of WNV infected mosquitoes in the population. Here, the MLE infection rate per 1000 specimens provides an estimate of the annual average number of WNV infected mosquitoes for each grid cell [40]. At the county scale, this MLE of the annual average number of WNV infected mosquitoes is highly correlated with observed annual human WNV human cases (*r* = 0.79, *P* < 0.001).

**Table 1:** Overview of human WNV cases and mosquito data in Suffolk County 2001–2015

Year	Human cases	Pools	Positive pools	Total mosquitoes	Trap locations	Vector abundance	Percent positive pools	WNV MIR	WNV MLE
2001	1	579	45	16,689	60	28.82	7.77	2.70	2.80
2002	8	546	20	12,332	71	22.59	3.66	1.62	1.65
2003	10	900	26	32,772	71	36.41	2.89	0.79	0.80
2004	0	462	7	13,720	43	29.70	1.52	0.51	0.51
2005	9	927	65	23,445	85	25.29	7.01	2.77	2.87
2006	2	699	52	23,276	45	33.30	7.44	2.23	2.33
2007	0	308	10	9077	35	29.47	3.25	1.10	1.12
2008	9	467	39	11,116	72	23.80	8.35	3.51	3.70
2009	1	660	14	21,074	35	31.93	2.12	0.66	0.67
2010	24	1289	276	34,701	82	26.92	21.41	7.95	9.06
2011	4	1173	67	45,833	57	39.07	5.71	1.46	1.51
2012	14	780	186	32,360	44	41.49	23.85	5.75	6.74
2013	4	1128	157	47,887	42	42.45	13.92	3.28	3.58
2014	1	1166	176	50,336	39	43.17	15.09	3.50	3.85
2015	5	1108	180	41,881	43	37.80	16.25	4.30	4.71

#### Meteorological and hydrological data

For this study we used meteorological variables extracted from the North American Land Data Assimilation Systems (NLDAS) project-2 for Suffolk County, Long Island. Hourly estimates of precipitation measured in millimeters per hour, temperature measured in Kelvin 2-m above ground, and specific humidity measured in kilograms per kilograms 2-m above ground were used to make monthly averages. Additionally we used Mosaic hydrology model simulations to estimate soil moisture content [41]. In particular, we used Mosaic model output layer one soil moisture (L1SM), which represents water content in the top 10 cm of the soil column, as a previous study found L1SM and another model output, root zone soil moisture (RZSM), which represents water content in the top 40 cm of the soil column, to be highly correlated [36]. The spatial resolution of both the NLDAS meteorological and Mosaic hydrological data is 0.125° (≈13 × 13 km grid cells) (Fig. 1). Aggregation of mosquito data by NLDAS grid cell discounts more local scale environmental factors that may bias trap collections and allows for analysis of how climate conditions influence relative mosquito infection rates.

#### Model data

In this analysis we used 15 years of surveillance data (2001–2015). The WNV infection rate was calculated for each of the 15 NLDAS grid cells in Suffolk County Long Island for which there were both meteorological and hydrological data available (Fig. 1). However, in some grid cells in certain years there were no surveillance data to calculate the WNV infection rate (*n* = 26). For grid cell 9 only two of 15 years had surveillance records, so we dropped this grid cell from further analysis.

### Statistical analysis

The statistical model (m09) used meteorological and hydrological conditions to estimate WNV pool infection rates in *Culex* mosquitoes in Suffolk County, Long Island, New York during 2001–2009 [36]. Briefly, a Poisson model with a dispersion parameter was used to model the annual percentage of *Culex* pools testing positive for WNV tallied for each NLDAS grid cell area. Regression was performed using combinations of meteorological (precipitation, temperature and specific humidity) and hydrological (RZSM or L1SM) monthly averages as the predictor variables. Combinations of predictor variables were restricted to only include one parameter of precipitation, temperature, and specific humidity each between January and August and one hydrological measurement of early season effects (January–April) and one of late season effects (May–August). The rationale behind restricting environmental predictor variables was to assess whether there was a connection between early season accumulation of standing water and later season drying, as tested by the two hydrological measurements.

Here we build on the m09 model using an expanded dataset (2001–2012) to explore alternate model forms and improve predictive performance. Several alternate model forms were tested, including negative binomial and hurdle. In contrast to the Poisson model, these alternate structures may provide more suitable forms for addressing over-dispersion and the hurdle model is better at modeling count data with many zero observations [42]. Shaman et al. (2011) noted a strong west–east gradient of meteorological and hydrological conditions, in particular that temperatures in spring and summer and hydrological conditions throughout the year are warmer and drier, respectively, in the western part of the county [36]. Here we account for this West–east gradient explicitly by using a mixed effects model with the location of the NLDAS grid cells as a random effect.

For each of the tested 2001–2012 (m12) model forms (Poisson, negative binomial, hurdle, mixed effects) we used the annual infection rate of WNV infection rate (hereafter referred to as the WNV infection rate) for each grid cell as the predictand. Regression was performed using all combinations (35,960) of meteorological (precipitation, temperature, and specific humidity) and hydrological (L1SM) monthly averages, January-August, as predictor variables. In contrast with the m09 model, we did not restrict combinations to one parameter of each climatic and hydrological variable. The large number of candidate models were tested for hypothesis generation as to the temporal importance of climatic and hydrological parameters. Monthly averages were restricted to January-August in order to precede or coincide with peak WNV infection in *Culex* mosquitoes. Best model form was identified based on whole model goodness-of-fit estimated using the Akaike Information Criterion (AIC).

Among the best-fitting models of the preferred model form, multimodel inference was used to identify the set of best-fitting models to be used to make parameter inferences and to calculate model-averaged predictions with unconditional confidence intervals [43]. Here we define ensemble modeling as the formal weighted averaging of simulations from multiple models, which is carried out in order to improve the overall accuracy of their competing predictions. To rank goodness-of-fit among the models tested, we calculated a second order AIC, AICc, which is a better estimation of model fit when the ratio of parameters to observations is small (n/k < 40). The Akaike weight, i.e. the weight of evidence of model *i* relative to the best model:

1 $$ {\omega}_i=\frac{ \exp \left(-\frac{1}{2}{\Delta}_i\right)}{{\displaystyle {\sum}_{i=1}^R} \exp \left(-\frac{1}{2}{\Delta}_r\right)} $$

was used to scale the relative plausibility of each fitted model given the data, where Δ_i_ = AICc_i_ – AICc_MIN_, AICc_MIN_ is the AICc of the best-fit model, and *R* is the number of models meriting inclusion. The inclusion criterion was the subset of models whose weights summed to 0.95 [44].

Model predictions were generated retrospectively for 2013, in real-time for 2014, and then retrospectively again for 2015. For the 2014 real-time predictions, we used available data, which necessitated prediction with a subset of possible model combinations. Specifically, for May, best-fitting models derived from only January-April meteorological and hydrological conditions were used to forecast the 2014 annual WNV infection rate for each grid cell. The same procedure was followed for each subsequent month with conditions from the associated temporal range: January though May for June predictions, January through June for July, January through July for August, and the full time period, January through August, for September.

The model-averaged prediction was determined as:

2 $$ \overline{\Theta}={\displaystyle {\sum}_{i=1}^R}{\omega}_i{\widehat{\varTheta}}_i $$

where Θ_*i*_ is the estimate of model *i.* The model-averaged variance was calculated using an unconditional estimator:

3 $$ Var\left(\widehat{\overline{\Theta}}\right)={\left[{\displaystyle {\sum}_{i=1}^R}{\omega}_i\sqrt{Var\left({\widehat{\Theta}}_i\Big|{g}_i\right)+\Big({\widehat{\Theta}}_i+}\widehat{\overline{\Theta}}\Big){}^2\right]}^2 $$

This unconditional estimator takes into account the variation within and between each model in the model set (i.e. the model selection uncertainty) and was used to estimate unconditional confidence intervals around each model-averaged prediction. Equations 1–3 were also used to develop multimodel parameter estimates.

We used the package glmmADMB to fit mixed effect models [45, 46] and MuMIn for model averaging [47]. All analyses were run in the statistical software R [48].

## Results

### Validation of M09

We first tested the predictive capability of the m09 statistical model, using meteorological and hydrological conditions to estimate WNV pooled infection rates in *Culex* mosquitoes in Suffolk County between 2001 and 2009 [36]. A comparison of modeled predictions for the next 5 years (2010–2015) with observed estimates of the percent of pools WNV positive resulted in a Root Mean Square Error (RMSE) of 15.77. The observations fall within the credible interval of the m09 predictions 95 % of the time but were not very accurate. This discrepancy warranted the testing of alternate model forms and multimodel inference as measures to improve the accuracy of predictions.

### Model selection

We used an expanded dataset of collections for 2001–2012 to explore alternate model forms and improve predictive performance. All model forms used the same set of environmental predictors (i.e. monthly estimates of precipitation, temperature, specific humidity, and soil moisture). Among the model forms tested, a mixed effects negative binomial (MENB) model with grid cell as a random effect resulted in the best fitting model by the Akaike Information Criterion, AIC. The MENB model was therefore exclusively used to develop predictions; however, even within this model form, the large number of parameter combinations tested resulted in a set of candidate models with equivocal fit. Furthermore, we found a wide range of predicted WNV infection rates between models (Additional file 1) indicating need for a formal probabilistic interpretation across predictions. Therefore, rather than pick the single best-fit MENB model, we used multimodel inference to develop ensemble predictions from the many MENB models. Table 2 presents the m12 model set, ranked by a second order AIC, AICc, which provides a better estimation of model fit than AIC when the ratio of parameters to observations is small. Based on their cumulative model weight, 16 models, each with four meteorological and hydrological parameters, were included in our multimodel inference and prediction.

**Table 2:** The M12 Model Set. The effects associated with each parameter, including the month used in the analysis, the parameter estimate, and in parentheses the standard error of the parameter estimate, are shown. The models included in the multimodel inference add to a weight of greater than or equal to 0.95. For this subset of models the weights have been rescaled to equal 1

Model rank	AICc	Weight	Temperature			Precipitation		Specific humidity		L1SM	
1	594.0	0.168	April 0.635 (0.111)	June 0.322 (0.093)		July -0.007 (0.003)		April -1255.622 (296.610)			
2	594.1	0.166	April 0.998 (0.091)			March 0.005 (0.001)	August 0.004 (0.001)	April -1993.274 (343.940)			
3	594.3	0.145	February −0.197 (0.046)	April 0.832 (0.095)		April -0.008 (0.002)		April -1169.784 (316.260)			
4	594.7	0.121	April 0.801 (0.094)			April -0.010 (0.002)		February -1244.152 (305.760)	April -999.791 (301.920)		
5	595.8	0.069	April 0.524 (0.086)			June 0.006 (0.002)				March 0.439 (0.083)	April -0.453 (0.079)
6	596.1	0.061	April 0.797 (0.083)			July -0.009 (0.003)		April -1607.247 (307.630)	June 476.888 (129.990)		
7	596.4	0.053	June 0.688 (0.090)	August 0.357 (0.121)		May 0.015 (0.005)		March 433.041 (132.250)			
8	596.6	0.046	April 0.298 (0.088)	June 0.480 (0.100)		April -0.006 (0.003)	June 0.006 (0.002)				
9	597.1	0.037	March 0.157 (0.045)	June 0.641 (0.097)	August 0.312 (0.126)	June 0.004 (0.002)					
10	597.5	0.029	June 0.727 (0.099)	August 0.528 (0.116)		January -0.013 (0.004)	May 0.022 (0.005)				
11	597.5	0.030	April 0.699 (0.107)					April -1057.684 (320.540)		March 0.313 (0.069)	April -0.323 (0.072)
12	598.3	0.019	May 0.848 (0.150)	July 0.314 (0.080)		April -0.011 (0.002)		May -1128.343 (245.040)			
13	598.4	0.019	June 0.668 (0.091)	August 0.389 (0.114)		April -0.007 (0.003)	May 0.019 (0.005)				
14	598.5	0.018	March 0.145 (0.045)	June 0.644 (0.093)	August 0.397 (0.119)	May 0.014 (0.005)					
15	599.5	0.011	April 0.522 (0.101)	June 0.352 (0.091)		April -0.005 (0.002)		April -1071.483 (294.770)			
16	599.8	0.009	April 0.430 (0.097)	June 0.408 (0.107)		March 0.004 (0.001)	June 0.009 (0.002)				

The parameters included in the m12 model set range in weighted importance (0.03–0.71) with a few key variables and a long tail of less important variables (Additional file 2). Focusing on the key parameters (those with weight above 0.1) we infer that the monthly meteorological parameters in April are the most important predictors of WNV infection rates in *Culex* mosquitoes. Specifically drier conditions in early spring (reduced precipitation and specific humidity and higher temperatures in April) lead to increased annual WNV infection rates in *Culex* mosquitoes.

### Multimodel inference

**Fig. 2:**
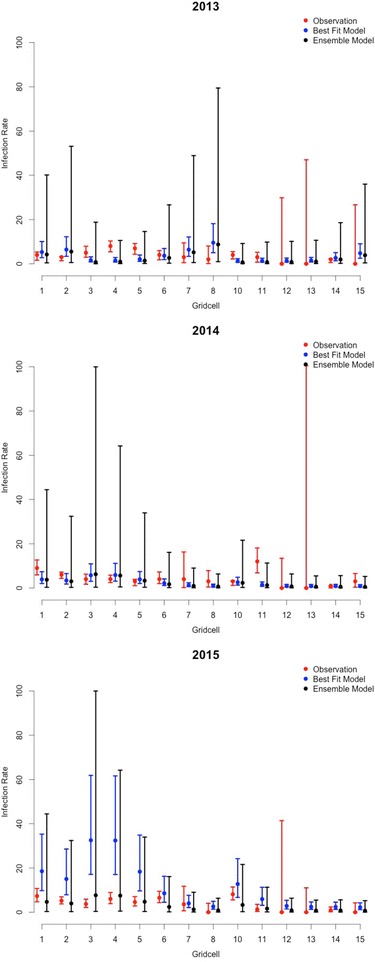
M12 Best Fit Model (*grey*) and Model-Averaged Predictions (*black*) with Unconditional Confidence Intervals for 2013–2014 and Observed Infection Rates and Confidence Intervals generated with PooledInfRate Excel Add-in [58] (*red*)

To make more stabilized predictions we averaged predicted values across component models based on the respective weight of each model and estimated the unconditional variance of the model-averaged prediction (See Eqs. 1 and 2 in Methods). The m12 ensemble model predictions had a much lower RMSE (3.90) than the predictions generated with the m09 model (RMSE = 10.11). The observations are within the m12 unconditional confidence intervals, indicating that overall the model weighted-average predictions are a good representation of the observations (Fig. 2). However, we note that the confidence intervals of the m12 ensemble model predictions are large relative to the low infection rates, reflecting sizeable, but accurately ascribed uncertainty, across the set of predictions.

### Predictions for 2014 in real-time

Model predictions for 2014 were generated in real time and changed with the inclusion of additional meteorological and hydrological estimates, as these data became available and the component model predictors were adjusted to accommodate that availability (see Methods). The January-April, January-May, and January-July time periods made almost identical predictions, while the January-June model made lower predictions and the January-August model made higher predictions for the western grid cells (1–6 & 10). All models generated consistently low predictions for the eastern grid cells (7–8 & 11–15).

For each time interval, we compared the number of component models and number of parameters that were included in the ensemble models as well as the RMSE illustrating the difference between predicted and observed WNV infection rates in 2014 (Additional file 3). We found that some of the lowest RMSE scores were for the earliest time periods suggesting, at least for 2014, predictions could be made early. Regardless of the time period selected the best fitting model included April temperature, precipitation, and specific humidity - with warm, dry April conditions favoring increased annual WNV infection rates in *Culex* mosquitoes - implying that early season meteorological conditions largely determine WNV infection rates.

### Leave-one-out temporal cross validation

**Fig. 3:**
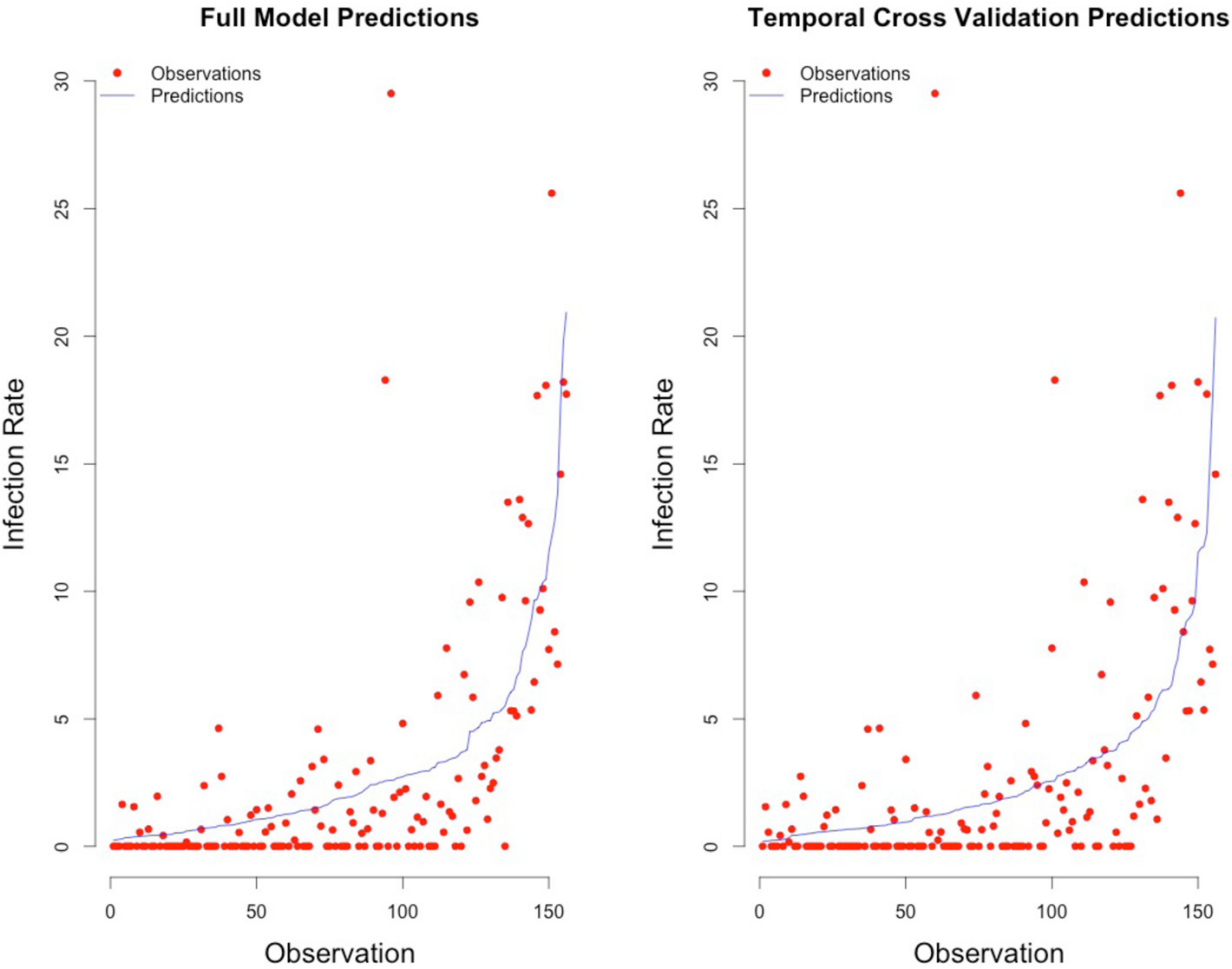
Temporal cross validation predictions and full model predictions. Graphs plot pairs of observations and predictions ordered by prediction value from least to greatest

Leave-one-out temporal cross validation (LOOTCV) was performed. Each year of data was iteratively omitted from the analysis and the compiled set of predictions from the LOOTCV models were then compared with predictions based on the full record. We found the LOOTCV model (RMSE = 4.27) and the full model (RMSE = 3.66) predictions comparable (Fig. 3), indicating that out-of-sample prediction (i.e. model predictions of a set of observations from a different time period) is possible and that no single year overly dominates the model structure. Figure 3 also illustrates that the m12 model is unable to distinguish among different low WNV infection rates (< 5 WNV infected mosquitoes per 1000) as these are predicted with nearly the same frequency. However, the model is able to tease apart differences in WNV Infection rates greater than 5 WNV infected mosquitoes per 1000 (Fig. 3). While the majority of observed infection rates are ≤ 5 WNV infected mosquitoes per 1000 (81 %), it is the remaining higher infection rates that are of greatest public health concern. The ability of the model to distinguish and predict these high infection events in both space and time is thus extremely valuable.

We tested the sensitivity and specificity of both the full model and LOOCV predictions. The sensitivity, or the probability that both the predicted and observed value are above 5, and the specificity, the probability that a predicted value fell below 5 while the observed value was below 5, were both high. For the full model the sensitivity is 94 % and specificity 85 %. For the LOOCV the sensitivity is 93 % and specificity 88 %.

These tests suggest that both the full and temporally cross-validated models are very good at distinguishing in both space and time high infection rates.

## Discussion

A central aim of this study was to assess the validity of using meteorological and hydrological conditions to predict WNV infection rates in *Culex* mosquitoes in Suffolk County. Model m09 [36] was used to make out-of-sample predictions for 2010–2015. Overall, these predictions captured the range and variability of observed values; however, individually, there were a number of predictions that failed to accurately estimate observations. This initial finding prompted the exploration of alternative model forms, as well as use of a set of models to make inferences. We found a MENB model to be the best model form and identified 16 component MENB models (m12), which we used to generate model-averaged predictions and unconditional confidence intervals. The m12 ensemble of models produced more accurate 2013–2015 predictions (RMSE = 3.90) than the m09 model (RMSE = 10.11). This finding indicates that weighted ensemble average predictions derived from an updated suite of best-fit models are a more informative and accurate forecast construct.

Although the predictors in the m12 and m09 models may at first seem contradictory, a closer look reveals they are actually well aligned. M09 findings indicated that wetter winter land surface conditions, warmer spring temperatures, increased spring precipitation, and drier early summer land surface conditions all favor the increased prevalence of WNV among *Culex* mosquitoes [36]. The m12 modeling effort indicates that less precipitation in April, more precipitation in May and June, along with warmer temperatures throughout the spring favor increased WNV activity in *Culex* mosquitoes. Although hydrological conditions were not identified as key parameters in the m12 model, they were included in two component models of the ensemble model and indicate that wetter March land-surface conditions favor WNV activity as for the best-fit m09 models. Unlike the m12 modeling effort, the m09 models were constrained to include a summer lag of L1SM (land surface wetness) conditions and thus found drier summer land surface conditions to be important as well. However, the m12 models did find that in addition to wetter March land-surface conditions, drier April land surface conditions favored WNV activity indicating a switch from wetland surface conditions early in the season to dry land surface conditions later in the season, similar to the findings of m09. The m12 model also indicates that reduced precipitation and specific humidity in April are important drivers of WNV infection rates emphasizing that dry April conditions are particularly important for WNV amplification – a result not specified by m09. However, like m09, m12 revealed an association between increased May precipitation and increased WNV infection rates, and both the m09 and m12 models identified an association between increased temperatures in spring and increased WNV infection rates.

Mechanistically, the pattern of wet conditions (in May and June) sandwiched between periods of drier conditions (in April and July) may provide the needed standing water conditions that then, during the dry summer conditions, become eutrophic, free of predators, and dense with avian hosts to elevate populations of infected *Culex* mosquitoes.

The climatic conditions identified here that promote WNV prevalence in mosquito vectors in Suffolk County, in particular warm temperatures and precipitation extremes, are becoming more common in the northeastern US [49]. The implications of these changing climatic conditions on WNV transmission risk are manifold: for mosquitoes, these conditions may elevate abundance and infection rates; for avian hosts, these conditions may alter migration, demography, and susceptibility; and for humans, these changes may confer increased transmission risk in mid to late summer.

Mosquitoes and the diseases they carry are climate sensitive. Mosquitoes need still water to breed, and temperature influences the rate of larval development, adult feeding behavior, and the rate of pathogen replication within the mosquito [10]. Because climactic factors influence the number of disease-infected mosquitoes, climate directly relates to the risk of disease transmission. Warm temperatures and increasing precipitation extremes may increase the risk of WNV; however, human manipulation of the environment, especially urbanization, and human behavior complicate the relationship between mosquitoes and climate. Impervious surfaces may change the hydrological conditions of an area and lead to water pooling and retention that may promote *Culex* mosquito breeding and increase the risk of spillover to humans where these habitats are proximal to people.

The arrival date of migrating birds and the beginning of the breeding season for resident birds is tied to spring temperatures and food availability [50–52]. Of the ten most competent WNV avian hosts in the US, eight are year-round residents of the northeast [53] and are adept at shifting their breeding behavior earlier in response to meteorological conditions [50]. *Culex pipiens* favor the American robin [54], which congregate in roosts throughout their breeding season. Roosting might make robins less available to *Culex* mosquitoes and result in *Culex pipiens* searching for alternative hosts and an associated increase of spillover transmission to humans in mid to late summer [7, 54, 55]. Should warmer spring temperatures effect still earlier arrival and roosting by robins, early enzootic amplification may be exacerbated.

In addition to climate, land use practices impact local ecology and WNV transmission. Avian diversity may reduce enzootic WNV transmission and spillover risk to humans [56, 57]. Bird diversity has declined in response to climate change [58], urbanization [59], and directly from WNV [60]. In comparison to residential areas, wetlands have lower enzootic WNV transmission tied to the higher avian diversity found in wetlands [44, 61]. Fragmentation due to urbanization can increase bird density [62]. Taken together, fragmentation of wetlands within residential zones may result in avian communities of reduced diversity, greater density, and therefore increased enzootic WNV amplification.

WNV amplification may be promoted through increased vector host contact rates or demoted through diluted vector host contact rates depending on the ratio of vectors to hosts. In roosts, higher numbers of competent avian hosts may reduce vector host contact rates and decrease exposure to infected mosquitoes in roosts [55, 63] but in combination with climatic conditions of reduced precipitation and increased temperature, the number of mosquito vectors may also increase and result in increased WNV amplification [9].

As in our study, other studies in the northeastern US have found that warmer than average temperatures influence WNV dynamics [19, 24, 28, 33, 35]. Winter temperatures may be a particularly important constraint on WNV dynamics in cold regions such as the northeastern US [22, 64] and future modeling efforts should include this winter effect. Lower then average annual precipitation has been associated with increased human WNV incidence [15] and vector abundance [33] in the eastern US. Results from Chicago also found that drought followed by wetting was associated with higher WNV infection rates in *Culex* mosquitoes in most years [21]. Our study, focused on a specific spatial location and not an entire region, is able to pinpoint when climate most strongly influences mosquito production and viral amplification in a given year.

The scale of the analysis can impact its outcome. The spatial resolution of the meteorological and hydrological data used in this study defined the spatial scale of the analysis, which was somewhat coarse and may obfuscate observation of patterns at a finer spatial scale. Furthermore, using monthly means of environmental conditions and annual estimates of WNV infection rates in *Culex* mosquitoes may limit temporal understanding. However, the goal of this analysis was to identify conditions predictive of high WNV infection rates in *Culex* mosquitoes indicative of increased WNV risk to humans in advance of this risk. Our results indicate that spring conditions are predictive of the annual intensity of WNV activity and how it varies across the county - information of high utility for vector control officers. Prediction of intra-annual variability, a more stochastic process, is a more challenging forecast problem; future research may need to make use of a mechanistic, process-based, modeling approach that depicts the transmission cycle and incorporates dynamic measurements of abiotic and biotic factors in order to generate accurate predictions of intra-annual variability in real time.

The multimodel inference used here addresses discrepancies between models of equivocal fit by defining a set of candidate models and making inferences based on the set rather than one best-fitting model. A comparison of the model-averaged predictions and the best fitting m12 model predictions suggests that the best fitting m12 model predictions are equivalent to the model-averaged predictions (*r* = 0.99) but with tighter confidence intervals principally due to the lack of model selection uncertainty (i.e. in this calculation there is only one model compared to 16 in the ensemble unconditional variance calculations) (Fig. 2). Given that the predictions vary substantially by model (Additional file 1) and that the evidence is not strong enough to warrant prediction with only one model (Table 2), the ensemble predictions and unconditional confidence intervals provide better inference. It is necessary to quantify uncertainty in model-generated predictions especially when communicating findings with vector control and public health personnel. While the uncertainty of the model-averaged estimates is large, these predictions exhibit high sensitivity and specificity for distinguishing high and low local WNV infection rates.

The utility of other modeling approaches, such as boosted regression trees, could be considered in future analyses. Due to the presence of open water throughout the study region, differences exist in the land area among grid cells. Future analysis may also want to explore a measure of infected mosquito density rather than abundance. Because our m12 findings corroborated the m09 findings, future modeling efforts should focus on the climatic conditions of April-July and determine the best climate predictors. This could be followed by investigation of whether the inclusion of other potential drivers (e.g. land use practices, distribution of wetlands) improves model performance. Incorporating these climatic and environmental drivers of WNV infection in *Culex* mosquitoes will inform our understanding of how future changes in climate will influence WNV amplification and transmission.

## Conclusions

In conclusion, few studies in the northeastern US have looked at the influence of climate on WNV vector infection rates, a measure that likely constitutes a more direct proxy of human WNV risk. In this study we validate a model built for prediction of WNV infection rates in *Culex* mosquitoes using meteorological and hydrological parameters. With additional years of surveillance, we develop an improved model form and use a set of best-fitting models to develop a multi-model prediction framework. The findings of the m09 and m12 models are aligned, with the m12 model emphasizing the importance of warmer, drier early spring (April) conditions for increasing WNV infection rates in *Culex* mosquitoes. This association allows prediction of annual *Culex* WNV infection rates early in the season, which can be used to inform vector control efforts both temporally, whether there will be particularly high WNV activity, and spatially, where WNV infection rates will be highest. Our study shows that real-time climate information can be used to make predictions before peak WNV infection in *Culex* mosquitoes and therefore before the risk of WNV transmission to humans is greatest.

## Additional files

Additional file 1: Variability of ensemble model predictions. Box plots showing the range of predictions across 16 ensemble models for each grid cell and year. The red dots indicate the observed WNV infection rate for that grid cell and year. (PDF 63 kb) Additional file 2: Ensemble Model Parameter Importance and Effect Size. Parameter Importance (left) and Coefficient Effect Sizes across all models in ensemble (right) together suggest that drier conditions in early spring lead to increased annual WNV infection rates in *Culex* mosquitoes. (PDF 93 kb) Additional file 3: Real-Time Ensemble Predictions for 2014. For 2014, some of the lowest RMSE scores were for the earliest time periods suggesting predictions could be made early. (XLSX 37 kb)
