# Supplementary figures and images for: Development and validation of a climate-based ensemble prediction model for West Nile Virus infection rates in Culex mosquitoes, Suffolk County, New York
Source: Parasit Vectors. 2016 Aug 9;9:443. doi: 10.1186/s13071-016-1720-1 (PMC4979155; doi:10.1186/s13071-016-1720-1)

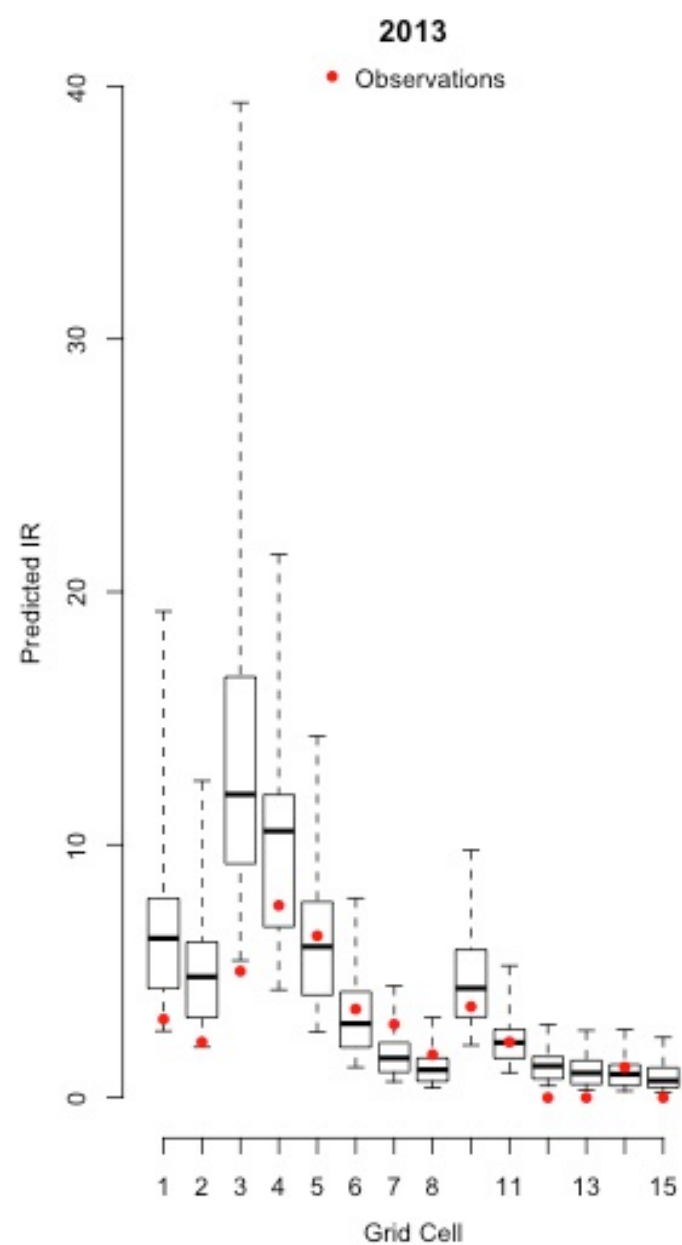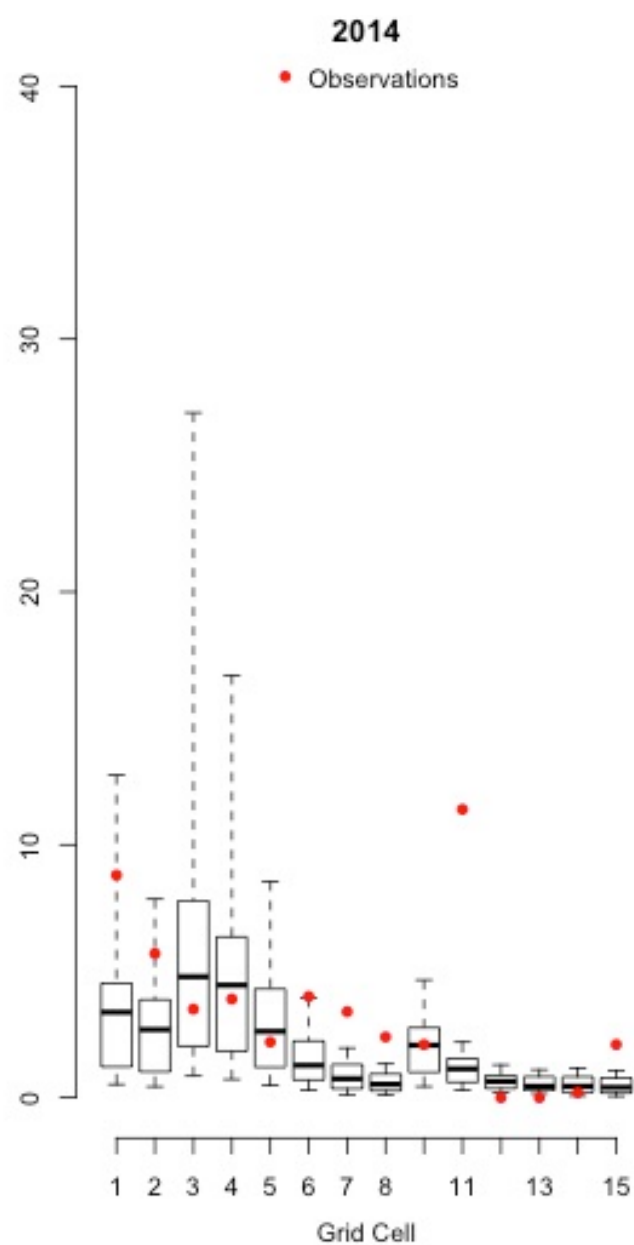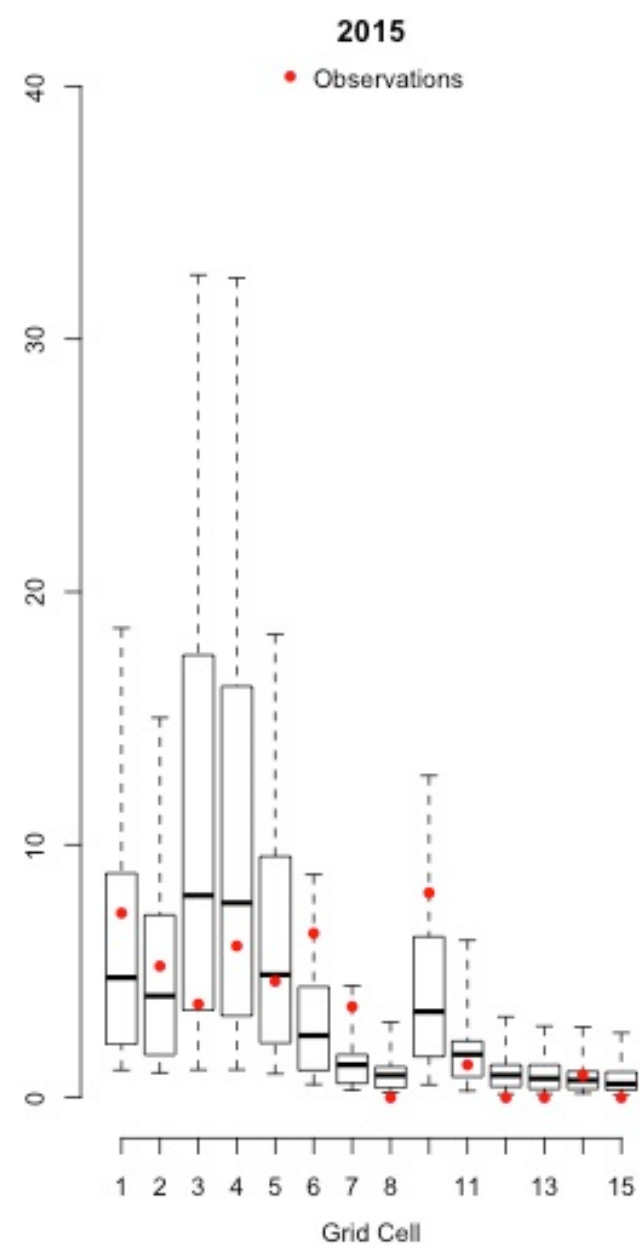

Supplement: Additional file 1: — Variability of ensemble model predictions. Box plots showing the range of predictions across 16 ensemble models for each grid cell and year. The red dots indicate the observed WNV infection rate for that grid cell and year. (PDF 63 kb) [file 13071_2016_1720_MOESM1_ESM.pdf]

## Parameter Importance

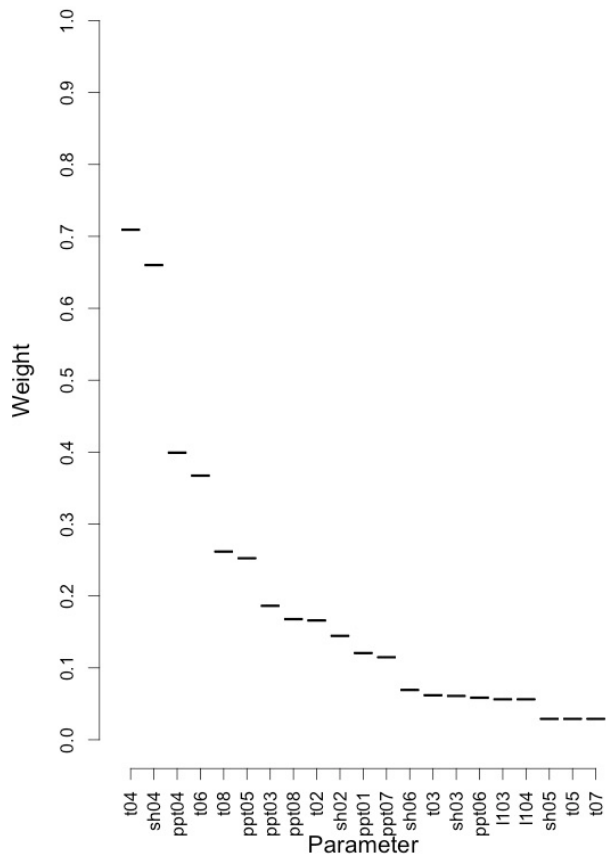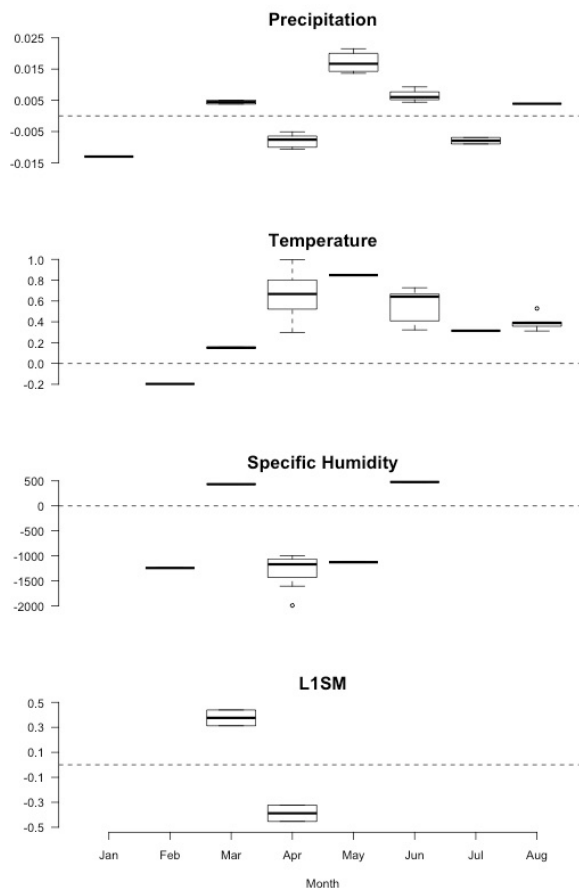

Supplement: Additional file 2: — Ensemble Model Parameter Importance and Effect Size. Parameter Importance (left) and Coefficient Effect Sizes across all models in ensemble (right) together suggest that drier conditions in early spring lead to increased annual WNV infection rates in Culex mosquitoes. (PDF 93 kb) [file 13071_2016_1720_MOESM2_ESM.pdf]
